# Analysis of Power Laws, Shape Collapses, and Neural Complexity: New Techniques and MATLAB Support via the NCC Toolbox

**DOI:** 10.3389/fphys.2016.00250

**Published:** 2016-06-27

**Authors:** Najja Marshall, Nicholas M. Timme, Nicholas Bennett, Monica Ripp, Edward Lautzenhiser, John M. Beggs

**Affiliations:** ^1^Department of Neuroscience, Columbia UniversityNew York, NY, USA; ^2^Department of Psychology, Indiana University - Purdue University IndianapolisIndianapolis, IN, USA; ^3^Department of Physics, Indiana UniversityBloomington, IN, USA; ^4^Department of Physics, Syracuse UniversitySyracuse, NY, USA; ^5^Biocomplexity Institute, Indiana UniversityBloomington, IN, USA

**Keywords:** neural criticality, neural avalanche, neural complexity, power law, shape collapse, information theory, MATLAB

## Abstract

Neural systems include interactions that occur across many scales. Two divergent methods for characterizing such interactions have drawn on the physical analysis of critical phenomena and the mathematical study of information. Inferring criticality in neural systems has traditionally rested on fitting power laws to the property distributions of “neural avalanches” (contiguous bursts of activity), but the fractal nature of avalanche shapes has recently emerged as another signature of criticality. On the other hand, neural complexity, an information theoretic measure, has been used to capture the interplay between the functional localization of brain regions and their integration for higher cognitive functions. Unfortunately, treatments of all three methods—power-law fitting, avalanche shape collapse, and neural complexity—have suffered from shortcomings. Empirical data often contain biases that introduce deviations from true power law in the tail and head of the distribution, but deviations in the tail have often been unconsidered; avalanche shape collapse has required manual parameter tuning; and the estimation of neural complexity has relied on small data sets or statistical assumptions for the sake of computational efficiency. In this paper we present technical advancements in the analysis of criticality and complexity in neural systems. We use maximum-likelihood estimation to automatically fit power laws with left and right cutoffs, present the first automated shape collapse algorithm, and describe new techniques to account for large numbers of neural variables and small data sets in the calculation of neural complexity. In order to facilitate future research in criticality and complexity, we have made the software utilized in this analysis freely available online in the MATLAB NCC (Neural Complexity and Criticality) Toolbox.

## 1. Introduction

Many recent studies have focused on evaluating self-organized criticality as a possible mechanism to explain neurological events (Beggs and Plenz, 2003, 2004). In general, these analyses seek to explain complex neurological data in terms of a relatively simple underlying system balanced at a critical point—a state between order and disorder. Many recent studies have produced evidence that suggests neural systems are poised at or near a critical point (Beggs and Plenz, 2003; Petermann et al., 2006; Mazzoni et al., 2007; Gireesh and Plenz, 2008; Pasquale et al., 2008; Hahn et al., 2010; Friedman et al., 2012; Priesemann et al., 2013, 2014; Williams-Garcia et al., 2014; Shew et al., 2015). Additional studies (see Beggs, 2008; Chialvo, 2010; Beggs and Timme, 2012 for reviews) have found important implications for the brain if it is indeed operating at or near a critical point, such as optimal communication (Beggs and Plenz, 2003; Bertschinger and Natschlager, 2004; Rämö et al., 2007; Tanaka et al., 2009; Shew et al., 2011), information storage (Socolar and Kauffman, 2003; Kauffman et al., 2004; Haldeman and Beggs, 2005), computational power (Bertschinger and Natschlager, 2004), dynamic range (Kinouchi and Copelli, 2006; Shew et al., 2009), and phase sychrony (Yang et al., 2012).

Research into criticality in neural systems has primarily focused on the analysis of contiguous sequences of neural activity, otherwise known as “neural avalanches” (Beggs and Plenz, 2003, 2004). In particular, a great deal of interest has concentrated on determining the existence of power laws in distributions of avalanche properties (see Beggs and Plenz, 2003; Priesemann et al., 2009; Shew et al., 2009; Klaus et al., 2011; Alstott et al., 2014; Ribeiro et al., 2014 as examples, Clauset et al., 2009; Touboul and Destexhe, 2010; Dehghani et al., 2012; Touboul and Destexhe, 2015 for critiques, and Beggs and Timme, 2012 for a review). However, recent research has expanded the number of analysis techniques (Beggs and Timme, 2012) to include shape collapse (Friedman et al., 2012; Priesemann et al., 2013), susceptibility (Williams-Garcia et al., 2014), and tuning through the critical point (Shew et al., 2009, 2011).

Research independent of neural criticality has attempted to develop methods to quantify the strength and nature of interactions between neurons across spatiotemporal scales (Tononi and Edelman, 1998; Tononi, 2004, 2008). One such information theoretic measure that captures the degree to which disjoint network components (e.g., individual neurons or groups thereof) coordinate their activity is referred to as “neural complexity” (Tononi et al., 1994). Neural complexity and similar measures have been suggested as diagnostic tools for various operational states of neural systems, including even consciousness (Tononi and Edelman, 1998; Tononi, 2004, 2008; Balduzzi and Tononi, 2008; Seth et al., 2011; Oizumi et al., 2014).

In this paper we present several significant improvements to criticality and complexity analyses. These improvements include: (1) We developed an automated maximum likelihood estimation (MLE) fitting routine for doubly truncated, discrete power-law distributions. This method allowed us to address sampling and finite-size effects in measuring power laws (Burroughs and Tebbens, 2001; Yu et al., 2014), as well as critiques of searching for power laws in neural data (Clauset et al., 2009; Touboul and Destexhe, 2010; Dehghani et al., 2012) by exclusively fitting the central part of the distribution. (2) We developed an automated method for performing and measuring avalanche shape collapses. This represents a significant improvement in methodology over previous manual shape collapses analyses (Friedman et al., 2012). (3) We developed automated methods to account for state sub-sampling in neural complexity calculations, thus improving the accuracy of these calculations in large systems of neural sources. (4) We made the software necessary to perform these analyses freely available in the MATLAB NNC (Neural Criticality and Complexity) Toolbox (see Supplementary Material, Timme, 2016).

The NCC Toolbox includes functions to carry out the operations discussed below, several programs for generating model data to test the software, as well as demo scripts to help new users explore the functionality of the programs. Other than the Statistics and Machine Learning Toolbox for MATLAB, the software is stand-alone and requires no additional libraries or functions. Given differences between individual analyses, the specific parameters discussed below can all be adjusted by the user either directly via explicit variables or via data preprocessing (e.g., applying initial data cuts). Importantly, though all of the analyses herein are discussed in terms of neural avalanches, most of the software and analysis methodologies can easily be applied to other types of data. For instance, the power-law fitting software can be applied to any type of probability distribution sample data. To aid the user in understanding the functionality of the software, throughout the paper we will present example code and information about data formats.

## 2. Background, methods, and models

### 2.1. Neural avalanches

**Figure 1:**
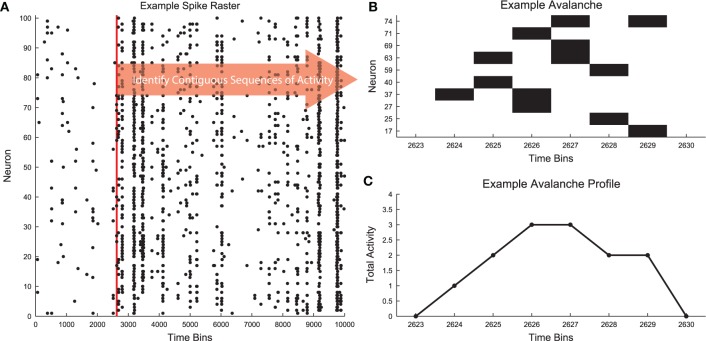
**Neural avalanches**. **(A)** A segment of the spike raster for all neurons in an example cortical branching model (see Section 2.3). **(B)** Example neuronal avalanche. Adjacent periods of activity were identified as avalanches. This avalanche corresponds to the red vertical line in **(A)**. This avalanche was duration 6 (6 time bins long) and size 13 (13 total neuron activations). **(C)** Avalanche profile for the example avalanche shown in **(B)**.

Neural avalanches are defined as sequences of time bins during which at least one neuron is active (Figure 1; Beggs and Plenz, 2003, 2004). Figure 1A contains an example segment of spiking data from a cortical branching model (see Section 2.3). The data clearly contain bursts of activity through the network. Figure 1B shows the spiking activity of several neurons that participated in a single avalanche. This avalanche had duration 6 because it involved six contiguous time bins with activity and it was size 13 because there were a total of 13 neuron activations during the avalanche. The shape or profile of the avalanche is then simply the number of active neurons at each time bin (Figure 1C).

### 2.2. Critical exponents and theoretical rationale for power laws

The study of critical phenomena in statistical mechanics provides concepts and notation that can be readily applied to neural avalanches (Sethna et al., 2001; Friedman et al., 2012). If a neural network operates near a critical point, then the size distribution (*f*_*s*_(*S*)), duration distribution (*f*_*d*_(*T*)), and average size given duration data (〈*S*〉(*T*)) of its avalanches can be fit to a power law (Equations 1–3).

(1) $$\begin{array}{r} f_{s}\left(S\right)\propto S^{-\tau } \end{array}$$

(2) $$\begin{array}{r} f_{d}\left(T\right)\propto T^{-\alpha } \end{array}$$

(3) $$\begin{array}{r} \langle S\rangle \left(T\right)\propto T^{1∕\sigma \nu z} \end{array}$$

In Equations (1–3), *S* is the size of an avalanche and *T* is the duration of an avalanche. The power-law exponents τ, α, and 1∕σν*z* are critical exponents of the system. They are model independent and identical for all systems in the same universality class (Sethna et al., 2001; Friedman et al., 2012).

### 2.3. Models

We employed several types of models to demonstrate our analysis improvements and the software functionality. First, we generated model data from various distributions in order to test the power-law fitting algorithm. Second, we used cortical branching models to produce neural avalanches to which the whole suite of analysis software could be applied. Third, we used a simplified version of the cortical branching model to provide intuitive examples that clarify the meaning of neural complexity.

We drew sample data from four types of distributions: power law, doubly truncated power law, exponential, and log normal. We generated 100 sample distributions from each model. Each sample was created by randomly assigning 10^5^ counts to the allowed discrete states (integers from 1 to 100) based on the normalized probability mass function (PMF) for that model. The unnormalized probability mass functions for the power-law (PL), doubly truncated power-law (TPL), exponential (E), and log-normal (LN) distributions are given by Equations (4–7).

(4) $$\begin{array}{lll} p_{PL}\left(x\right) & =x^{-\tau }\; & \; \end{array}$$

(5) $$p_{TPL}(x)=\{\begin{array}{ll} e^{-\lambda x}\frac{x_{min}^{-\tau }}{e^{-\lambda x_{min}}} & 1\leq x<x_{min}\\ x^{-\tau } & x_{min}\leq x\leq x_{max}\\ e^{-\lambda x}\frac{x_{max}^{-\tau }}{e^{-\lambda x_{max}}} & x_{max}<x\leq 100 \end{array}$$

(6) $$\begin{array}{lll} p_{E}\left(x\right) & =e^{-\lambda x}\; & \; \end{array}$$

(7) $$\begin{array}{lll} p_{LN}\left(x\right) & =\frac{e^{-{\left(\log\left(x\right)-\mu \right)}^{2}∕\left(2\sigma^{2}\right)}}{x}\; & \; \end{array}$$

For all distribution models, τ = 2.5, σ = 2, λ = 0.125, μ = 0.3, *x*_*min*_ = 10, and *x*_*max*_ = 75. Each distribution was normalized to the range of discrete values considered (1 to 100) via Equation (8).

(8) $$\begin{array}{l} p_{norm}\left(x\right)=\frac{p\left(x\right)}{\overset{100}{\underset{i=1}{\sum }}p\left(i\right)} \end{array}$$

In addition to the distribution models, we used a cortical branching model to produce neural avalanches that we then analyzed using the entire software package (Haldeman and Beggs, 2005; Williams-Garcia et al., 2014). The cortical branching model contained 100 neurons arranged in a square lattice with periodic boundary conditions (i.e., a torus). The model utilized a spontaneous firing probability to randomly create activity in the network. At each time step, each neuron had a $p_{spont}=10^{-4}$ likelihood to spike spontaneously. Activity in the networks propagated via interactions between a neuron and its four nearest neighbors. If a given neuron spiked at time *t*, there was a *p*_*trans*_ likelihood that it would cause one of its neighbors to spike at time *t* + 1. For demonstration purposes in this analysis, we used *p*_*trans*_ = 0.26. We chose a value of *p*_*trans*_ ~ 1 ∕ 4 to produce sustained periods of activity because each neuron had four neighbors. The model was run for 3 ^*^ 10^5^ time steps and produced 2794 avalanches. In this model, we know the appropriate time scale at which the data should be analyzed (one time bin equals one time step). However, in analyses of biological data, the optimal bin size is not known and the bin size can dramatically affect the criticality analysis. Specifically, larger bins tend to group temporally proximal avalanches together, while smaller bins tend to fragment or completely destroy small avalanches. Therefore, special attention should be paid to bin size when working with biological data. One possible solution is to use the average interspike interval (for neuron spiking data) as an estimate of the time scale for the system, though other methods may be more appropriate given the situation.

While the full cortical branching model was capable of generating neural avalanches, we used a simplified model to generate spiking activity with varying degrees of complexity. This simplified model consisted of 12 neurons arranged in a feedforward chain (i.e., neuron 1 could influence neuron 2, but not vice versa). All interactions in the network were instantaneous and the neurons had no refractory period. The spiking state of neuron 1 alternated at each time step. The spiking state of the *i*^*th*^ neuron (call it *a*_*i*_) was coupled to the spiking state of the (*i* + 1)^*th*^ neuron (call it *a*_*i*+1_) using a parameter *c* such that *p*(*a*_*i*+1_ = *a*_*i*_) = 0.5(1 + *c*) and *p*(*a*_*i*+1_ ≠ *a*_*i*_) = 0.5(1 − *c*). Therefore, the system produced totally random data for *c* = 0 and totally ordered data for *c* = 1. In this analysis, we utilized values of *c* = 0, 0.8, 1 to probe different levels of complexity.

### 2.4. Software

The NCC MATLAB toolbox (see Supplementary Material, Timme, 2016) contains an example neural avalanche data set generated by the cortical branching model (sample_data.mat, see Section 2.3) that was analyzed to produce all figures that utilize cortical branching data throughout the paper. These data are stored in a novel data format referred to as asdf2 (Another Spiking Data Format version 2), which utilizes Matlab structures. The fields of the structure are described in Table 1. Data in the asdf2 format can easily be rebinned to large bin sizes using the function rebin:

**» asdf2 = rebin(asdf2,4); % Rebin from 1 ms bins
          to 4 ms bins**

**Table 1:** **asdf2 data format fields**.

**Field**	**Class**	**Value**
binsize	double	Size of the time bins in milliseconds
nbins	double	Number of time bins in the recording
nchannels	double	Number of recorded channels (e.g., electrodes or neurons)
expsys	string	Type of experimental system
datatype	string	Data type (e.g., “spikes” or “LFP”)
dataID	string	Experiment specific identifier
raster	cell array	Spike or event times for each channel as a double vector

Furthermore, data in the asdf2 format can be converted to raster format using the function asdf2toraster and the inverse operation can be performed using rastertoasdf2:

**» raster = asdf2toraster(asdf2);
» asdf2 = rastertoasdf2(raster, binSize, expSys,
          dataType, dataID);**

A spike raster is a double array in which each row corresponds to a channel (e.g., neuron) and each column to a time bin. For a given channel and bin, the presence or absence of a spike is indicated by a one or zero, respectively. The raster data format is primarily used in the complexity analysis (see Section 5.4).

Data in the asdf2 format can easily be converted to avalanches using the function avprops:

**» Av = avprops(asdf2);**

The Av variable is a structure with three fields. Av.duration is a double vector that records the duration of each avalanche. Av.size is a double vector that records the size of each avalanche. Av.shape is a cell array that contains the avalanche shape (number of active sites during each time step of the avalanche). Note that Av.duration(i), Av.size(i), and Av.shape{i} all refer to the same avalanche and that the avalanches are ordered in time. The function avprops is also capable of calculating a simple branching ratio estimate (Haldeman and Beggs, 2005) and the fingerprint of the avalanches (i.e., the shape with information about which channels were active during a given time bin).

Several functions are included in the toolbox to generate model data as described in Section 2.3. The function gendata can be used to generate random discrete data over a defined interval following power-law, truncated power-law, exponential, log normal, or exponentially-modified power-law distributions. In addition, gendata can generate random continuous data for power-law or exponential distributions.

**» % 10000 samples from a power-law distribution:**
  *p*(*x*) α *x*^−2.5^
**» x = gendata(10000, {’powerlaw’, 2.5});
» % 1000 samples from an exponential
  distribution:** *p*(*x*) α *e*^−0.125*x*^
**» x = gendata(1000, {’exponential’, 0.125});**

Data from these distributions can be generated with variable numbers of samples, model parameters, and maximum or minimum values. While gendata produces data with random fluctuations, the function pldist can be used to produce perfectly power-law discrete data with various types of truncation and slopes, primarily for software testing purposes:

**» x = pldist(1000);**

Cortical-branching-model data can be generated with the function cbmodel:

**» % A 100 node network with a transmission
probability of 0.26
» asdf2 = cbmodel(0.26);**

Data from the cortical branching model can be generated using variable numbers of neurons, spontaneous activity probabilities, and recording lengths.

## 3. Power-law fitting

### 3.1. Background

Power-law or power-law-like distributed data have been observed in a wide range of contexts, including neuroscience phenomena such as neural network degree (Bonifazi et al., 2009; Lorimer et al., 2015) and neural avalanche size (Beggs and Plenz, 2003), as well as phenomena outside of neuroscience such as terrorist attack deaths and solar flare intensity (Clauset et al., 2009). Therefore, the subject of power-law fitting in general (Burroughs and Tebbens, 2001; Goldstein et al., 2004; Perline, 2005; White et al., 2008; Clauset et al., 2009; Priesemann et al., 2009; Holden and Rajaraman, 2012; Deluca and Corral, 2013) and power-law fitting in analyses of neural criticality in particular (Priesemann et al., 2009; Touboul and Destexhe, 2010; Klaus et al., 2011; Dehghani et al., 2012; Alstott et al., 2014; Ribeiro et al., 2014; Yu et al., 2014; Touboul and Destexhe, 2015) have received a great deal of attention in the literature. Fitting power laws has proven to be a difficult task, though recent studies have greatly improved power-law fitting methodology (Goldstein et al., 2004; White et al., 2008; Clauset et al., 2009; Priesemann et al., 2009; Deluca and Corral, 2013; Alstott et al., 2014). Unfortunately, one outstanding issue in the literature is accurately fitting doubly truncated, discrete power-law distributions (i.e., distributions with a minimum and a maximum cutoff; see Deluca and Corral, 2013 for a treatment of continuous distributions and (Clauset et al., 2009) for a treatment of distributions with a minimum cutoff). This issue is significant because the vast majority of real potentially power-law data are doubly truncated. The maximum cutoff is typically caused by finite size effects. In other words, there are often practical limits to the largest data that can be measured (e.g., a limit to the maximum number of neurons that can be recorded simultaneously or even the maximum number of neurons in the brain). Previous methods do not incorporate these realities of experimental data and instead apply power-law fits with only minimum cutoffs (e.g., Clauset et al., 2009) or require the user to manually set the maximum cutoff (e.g., Alstott et al., 2014; Yu et al., 2014).

One might assume that neglecting the existence of a maximum cutoff would simply produce noise in the tail of the distribution that would be incorporated by the fitting procedure, however the consequences are in fact more severe. Previous power-law fitting methods (see Clauset et al., 2009 for the method and Dehghani et al., 2012 for an example in neural systems) rely heavily on cumulative distribution functions (CDFs) for fitting and plotting in addition to probability density functions (PDFs). This is not problematic for power-law distributions with only a minimum cutoff because those distributions produce power-law CDFs. Indeed, plotting CDFs can be beneficial in terms of avoiding bias associated with binning and noise. However, doubly truncated power-laws distributions do not produce power-law CDFs (Burroughs and Tebbens, 2001; Yu et al., 2014). Rather, the CDFs of doubly truncated power laws appear bent. So, using the previous methodology with doubly truncated power laws (i.e., nearly all real data) will produce inaccurate results and CDF plots that are obviously not power law (Burroughs and Tebbens, 2001; Dehghani et al., 2012; Yu et al., 2014; see **Figure 4** below for an example). This is the case even when the data are truly power law, but simply possess a maximum cutoff in addition to a minimum cutoff. The methodology we introduce herein addresses these concerns for discrete power-law data.

### 3.2. Improvements

Similar to Yu et al. (2014), we extended previously used MLE techniques to doubly truncated, discrete power laws (Clauset et al., 2009; Deluca and Corral, 2013). Before describing how we applied the power-law fitting algorithm to the data, we will first describe the power-law fitting itself.

For a truncated, discrete power law, the probability mass function is given by Equation (9).

(9) $$\begin{array}{l} f\left(x\right)=A\left(\alpha ,\;x_{min},\;x_{max}\right){\left(\frac{1}{x}\right)}^{\alpha } \end{array}$$

In Equation (9), *A* is a normalization constant, {*x* ∈ ℤ:*x*_*min*_ ≤ *x* ≤ *x*_*max*_}, {α ∈ ℝ:α > 1}, *x*_*min*_ is the minimum *x*-value, and *x*_*max*_ is the maximum value. The value of *A* can be found by normalizing the probability mass function (Equation 10).

(10) $$\begin{array}{l} A\left(\alpha ,\;x_{min},\;x_{max}\right)=\frac{1}{\overset{x_{max}}{\underset{x=x_{min}}{\sum }}{\left(\frac{1}{x}\right)}^{\alpha }} \end{array}$$

The likelihood function *L*(α) for all *N* of the individual *x*_*i*_ measurements is given by Equation (11).

(11) $$\begin{array}{l} L\left(\alpha \right)=\overset{N}{\underset{i=1}{\prod }}f\left(x_{i}\right)=A{\left(\alpha ,\;x_{min},\;x_{max}\right)}^{N}\overset{N}{\underset{i=1}{\prod }}{\left(\frac{1}{x_{i}}\right)}^{\alpha } \end{array}$$

Then, the log-likelihood is given by Equation (12) (including division by *N*).

(12) $$\begin{array}{lll} l\left(\alpha \right) & =\frac{1}{N}\log\left(L\left(\alpha \right)\right)\; & \;\\ =\frac{1}{N}\left(N\log\left(A\left(\alpha ,\;x_{min},\;x_{max}\right)\right)+\overset{N}{\underset{i=1}{\sum }}\log\left({\left(\frac{1}{x_{i}}\right)}^{\alpha }\right)\right)\; & \;\\ =-\log\left(\overset{x_{max}}{\underset{x=x_{min}}{\sum }}{\left(\frac{1}{x}\right)}^{\alpha }\right)-\frac{\alpha }{N}\overset{N}{\underset{i=1}{\sum }}\log\left(x_{i}\right)\; & \; \end{array}$$

Note that the first expression on the last row of Equation (12) is monotonically increasing in α, while the second term is linearly decreasing in α. Therefore, there will be a unique maximum value for *l*(α). We used a lattice search algorithm to estimate the power-law exponent α_*fit*_ that maximized *l*(α).

The lattice search functioned by first calculating *l*(α) for the interval 1 ≤ α_*fit*_ ≤ 5 at increments of 0.1. It then selected the value of α_*fit*_ that produced the largest value of *l*(α) (call this α_*fit*, 1_). It then calculated *l*(α) over the interval α_*fit*, 1_ − 0.1 ≤ α_*fit*_ ≤ α_*fit*, 1_ + 0.1 at increments of 0.01. If α_*fit*, 1_ was equal to 1 or 5, the bound for the new iteration was set as α_*fit*, 1_. The algorithm then selected the value of α_*fit*_ that produced the largest value of *l*(α) (call this α_*fit*, 2_). The algorithm continued until a precision of 10^−3^ was reached (See Section 3.4 for an example test of this search process in comparison to methods supplied by Clauset et al., 2009).

We will now discuss the details of how we applied this fitting tool to the neural avalanche data, though we wish to emphasize that these same analysis methods could be applied to any type of power-law data. In neural avalanche analyses, we will be interested in fitting the distributions of avalanche sizes and durations (see Section 2.1). Prior to fitting the distributions, we applied cuts to the data. For a given type of distribution (size or duration), we removed avalanches with sizes or durations less than 4 as well as data for which less than 20 avalanches of that size or duration were observed. These cuts were imposed in order to consider similar portions of the data in the power-law fit analysis as we considered in the shape collapse analyses (see Section 4.2). Note that because the fitting method can account for doubly truncated data, removing data from the left and right portion of the distribution via these cuts does not bias the fitting as would be the case with methods that do not account for double truncation.

**Figure 2:**
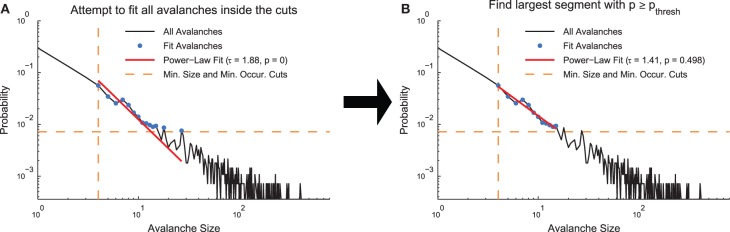
**Power-law fit search algorithm**. **(A)** A MLE fit is performed on the avalanches after minimum size or duration cuts and occurrence cuts are applied. **(B)** If the *p* < *p*_*thresh*_ in **(A)**, progressively smaller ranges of avalanche sizes or durations are fit until *p* ≥ *p*_*thresh*_. The data for this explanatory diagram was taken from the cortical branching model.

Following the application of cuts to a given distribution, we used the MLE fitting algorithm discussed above to estimate the truncated power law that best fit the distribution. While this algorithm always produced the best fit in the MLE sense, it was not always the case that this fit was acceptable. Therefore, we used the following algorithm to quantify acceptable fits (Clauset et al., 2009; Deluca and Corral, 2013) (Figure 2). We used a power-law model to produce data sets (*N*_*PLM*_ = 500 model data sets) over the fit range and compared the KS-statistics between (1) the real data and the fit against (2) the model data and the fit. If the real data produced a KS-statistic that was less than the KS-statistic found for at least 20% of the power-law models (i.e., *p* ≥ *p*_*thresh*_ = 0.2), we accepted the data as being fit by the truncated power law because the fluctuations of the real data from the power law were similar in the KS sense to random fluctuations in a perfect power-law model. However, if the converse was true, we rejected the truncated power-law hypothesis. Note that this method is not able to prove the data were generated by a truncated power law, rather it is only able to reject the truncated power-law hypothesis (see Section 3.3). In order to decrease computation time in computing the acceptance criterion, we terminated model generation if the likelihood of success (*p* ≥ *p*_*thresh*_) fell below 0.1% as determined by a binomial CDF under the assumption the likelihood of success (i.e., finding a model data set with KS-statistic larger than for the real data) was at the minimum value (i.e., *p* = *p*_*thresh*_).

If the truncated power-law hypothesis was rejected, we searched for successively smaller ranges of the distributions that could be fit by the truncated power law using the same methodology discussed above. We defined the ranges in a logarithmic sense using *range* = *log*(*size*_*max*_)∕*log*(*size*_*min*_) for size distributions and *range* = *log*(*dur*_*max*_)∕*log*(*dur*_*min*_) for duration distributions. Once a range was found over which the truncated power-law hypothesis was accepted, we ceased the search. Because the algorithm searched through successively smaller fit ranges, the fit ranges reported by the analysis represent the largest segment of the data that was fit by a truncated power law.

**Figure 3:**
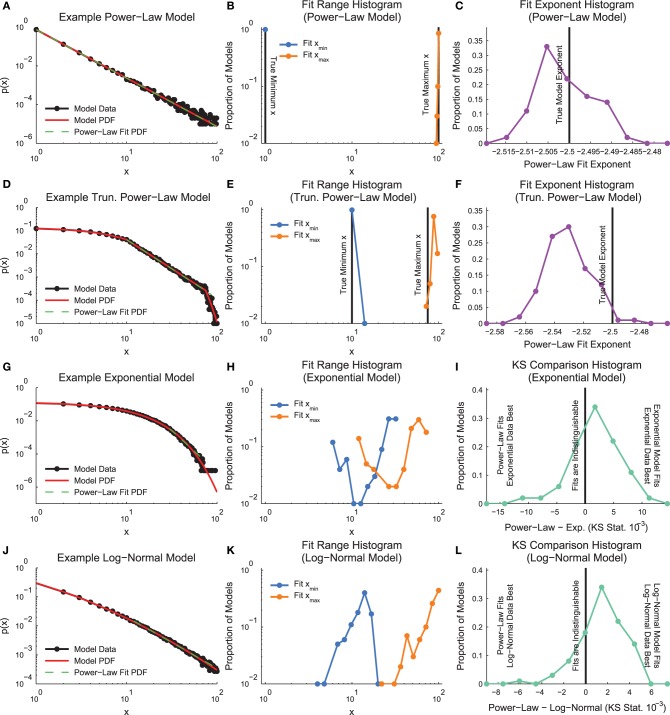
**Model distribution power-law fit results**. **(A, D, G, J)** Example model distributions. Note that the truncated power-law search algorithm finds power-law segments of all four models. **(B, E, H, K)** Histograms of minimum and maximum power-law fit regions. Note that the fit method finds the power-law segment of the truncated power-law model, though it does tend to overestimate the fit range. **(C,F)** Histogram of fit exponents for the power-law and truncated power-law models. The fit finds the power-law exponent with very small error, but it tends to slightly underestimate the truncated power-law exponent **(I,L)** KS-statistic comparison between power-law fits and the real model. Note that for these fits, a large proportion of the data sets are better fit by a truncated power-law distribution than the non-power-law distribution that was used to generate the model data.

In order to demonstrate how the fitting algorithm handles different types of data, we applied the MLE truncated power-law fitting method to four types of discrete model distributions (see Section 2.3, Figure 3): power law, truncated power law, exponential, and log normal.

An example power-law model is shown in Figure 3A. The fitting algorithm typically fit the whole range of the distribution (0–100; Figure 3B). The fitting algorithm produced exponents very near the exponent from the distribution used to create the models (Figure 3C).

An example truncated power-law model is shown in Figure 3D. The fitting algorithm automatically detected fit ranges very close to the true range of the power-law segment of the data, though the algorithm did tend to slightly overfit near the end of the distribution (Figure 3E). Due to the slight overfitting at the end of the distribution and the downward curve in the data following the power-law segment, the fitting algorithm found power-law exponents very close to the real values, though with a slight downward bias (Figure 3F).

An example exponential model is shown in Figure 3G. The fitting algorithm tended to identify short segments in the tail of the distribution as being power-law (Figure 3H). The fitting of small segments of non-power-law data using a power-law function was caused by the proximity in a KS-statistic sense between the fit and the model data (Figure 3I).

An example log-normal model is shown in Figure 3J. Similarly to the exponential models, the log-normal models tended to be fit as power law in segments of the tail due to the proximity between the model data and the power-law fit in a KS-statistic sense (Figures 3K,L).

**Figure 4:**
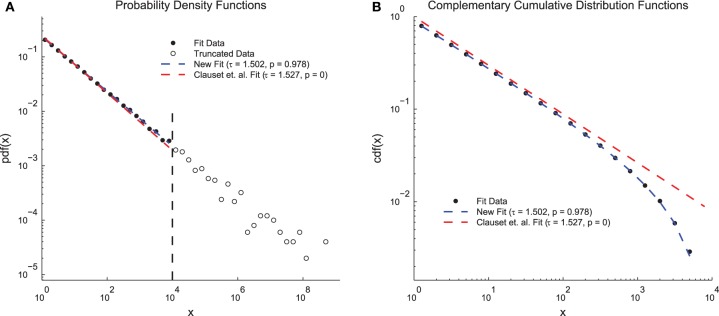
**Clauset et. al. power-law fit method comparison**. **(A)** Probability density functions for truncated simple model data, a fit using the methods introduced herein, and a fit using previous methods (Clauset et al., 2009). Both methods produce fits that qualitatively match the data and that have exponents near the true value of τ = 1.5. However, the previous methods produce a low *p*-value (reject power-law hypothesis) and the new methods produce a high *p*-value (accept power-law hypothesis). **(B)** Complementary cumulative distribution functions for the fit data from **(A)**. The CDF of the model data appears bent due to the truncation. The new methods introduced herein are able to account for this distortion and still fit the data. The previous methods produce a low *p*-value because they assume the CDF will still be a power law.

In order to demonstrate the advantages of the fitting techniques introduced herein, we compared the performance of the new fitting techniques on a simple data set to the performance of a previous method that does not include double truncation (Clauset et al., 2009; Figure 4). We generated continuous power-law distributed data (τ = 1.5 and *N* = 50, 000) and artificially truncated the data at 10^4^. When plotted as PDFs (Figure 4A), both methods produced fits that qualitatively match the data and yielded fit exponents near the true value. However, the previous methodology produced a *p* = 0 (indicating the power-law hypothesis was rejected) and the new methodology produced a *p* = 0.978 (indicating the power-law hypothesis was accepted). These results can be explained by examining the CDFs (Figure 4B). The CDF of the fit data appears bent because of the truncation (Burroughs and Tebbens, 2001; Yu et al., 2014) and only the CDF from the new method fit takes this distortion into account. Because the *p*-value calculation relies on the CDF, accounting for this type of truncation is vital for an accurate measurement of the *p*-value. This problem could be further compounded if only the CDF were plotted (e.g., Dehghani et al., 2012) because then even the qualitative agreement between the fit and the PDF would be missed.

**Figure 5:**
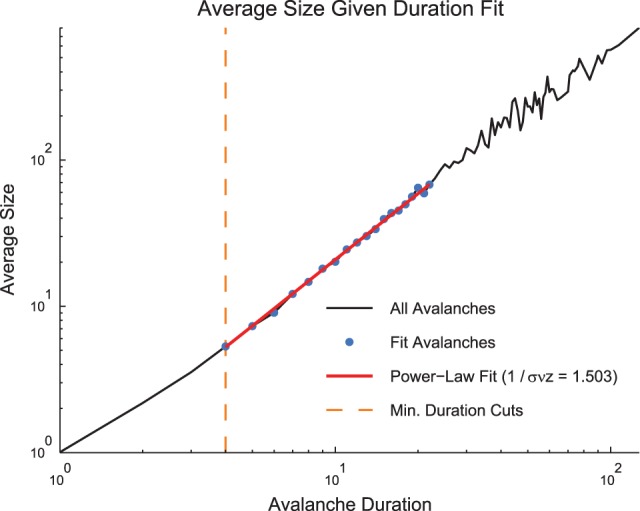
**Size given duration fit**. The average avalanche size as a function of avalanche duration follows a power law with a positive slope. Because these data are not a probability distribution, an MLE approach to fitting was not possible. We used a weight least squares fitting of the logarithmically scaled durations and average sizes. We also preserved minimum duration and occurrence cuts from the MLE fitting approach for size and duration distributions. Note, using the average size given duration fit, we found 1∕σν*z* = 1.503. Using the shape collapse analysis, we found 1∕σν*z* = 1.498 (Figure 6E). This represents a difference of 0.3%. The data for this figure was taken from the cortical branching model.

In addition to fitting the size and duration distributions with truncated power laws, we also wished to fit the values of the average avalanche sizes given duration for each data set (Figure 5). These data are also hypothesized to follow a power law (see Section 2.2). However, unlike the size and duration distributions, the average size given duration plots show power laws with positive exponents. This is expected since long duration avalanches, while less likely than short duration avalanches, are more likely to have a larger size than short duration avalanches. Because the average size given duration data is not a probability distribution, we were unable to fit it using an MLE approach. Instead, we used a simple weighted least squares fitting algorithm via the standard Matlab function lscov. We logarithmically scaled the durations and average sizes, and we used the number of avalanches of a given duration as the weight. We only fit size given duration data for avalanches that fell in the duration range fit by a truncated power law using the methods discussed above.

### 3.3. Discussion

The subject of power-law fitting in analyses of neural criticality has been controversial in the literature (Priesemann et al., 2009; Touboul and Destexhe, 2010; Klaus et al., 2011; Dehghani et al., 2012; Alstott et al., 2014; Ribeiro et al., 2014; Yu et al., 2014; Touboul and Destexhe, 2015). As we have noted previously, the existence of a power law does not prove a system is critical (Beggs and Timme, 2012). Other phenomena can generate power laws and experimental concerns can obscure power laws (e.g., limits to data gathering Stumpf et al., 2005). That having been said, the frequent appearance of power laws in neurological data is noteworthy and requires explanation. One possible explanation is that the underlying system is operating at or near a critical point, thus making further analyses related to criticality (e.g., shape collapse analyses) necessary. The fact that other beneficial qualities are associated with operating at a critical point (e.g., optimal information processing Beggs and Timme, 2012) makes additional criticality studies all the more important.

Beyond those concerns which motivate our study of criticality and our response to studies that are critical of the criticality hypothesis, there are several technical concerns related to power-law fitting that require comment. Recent studies have greatly improved power-law fitting methodology (Goldstein et al., 2004; White et al., 2008; Clauset et al., 2009; Priesemann et al., 2009; Deluca and Corral, 2013; Alstott et al., 2014). In our analysis, we continued these advances by introducing an automated MLE technique to fit doubly truncated discrete power-law distributions. This represents a significant advance in power-law fitting because the vast majority of experimentally gathered data is doubly truncated.

While our methodology represents an important advance in power-law fitting, we wish to emphasize that it does not address an important issue. As we showed above (Figure 3), our methodology finds power-law portions of non-power-law distributed data. In other words, our curve fitting routine allows for data that diverges from power law at the top and bottom of a distribution in order to account for finite size effects and other common data analysis complications. However, it is not always clear when this allowance is justified to correct for data gathering limitations in truly power-law data or when this allowance will result in simply fitting a small segment of non-power-law data as being power law. Fundamentally, this error results from fact that our power-law fitting algorithm is only able to reject a power-law fit. It cannot confirm that a power-law function generated the data, nor could any other fitting algorithm. When our algorithm accepts a segment of a distribution as being generated by a power law, the algorithm is making a statement that the deviation in the data from the power-law fit is below a certain threshold. When our algorithm does not accept a segment of a distribution as being generated by a power law, the algorithm is rejecting the power-law hypothesis, similar to how other statistical tests reject null models when low *p*-values are found.

To account for this fit acceptance phenomena, it is vital to compare the fit to some null or alternative model, so a statement can be made as to whether the data are more or less power law than a null or alternative model. Such models could be generated by randomizing the original data or via some other known distribution (e.g., exponential, log-normal, etc.; Clauset et al., 2009; Alstott et al., 2014). The null or alternative model should be selected with care for several reasons. First, if an alternative model is chosen with several adjustable parameters, it is possible that the alternative model will fit the data better than a power law simply because of the additional freedom supplied by the adjustable parameters. Methods exist to aid in comparisons between models with different numbers of parameters (Akaike, 1998) and these should be consulted when performing a comparison with an alternative model. Furthermore, biases in the data gathering methods (e.g., finite size effects) may bias certain regions of the distribution in complex ways, which may hamper attempts to create the appropriate null model. If randomized versions of the original data are desired, several methods exist including swapping and jittering to create randomized data (Rolston et al., 2007).

In principle, it could be possible to utilize similar techniques to those used above and elsewhere (Deluca and Corral, 2013) to develop doubly truncated automated MLE fitting algorithms for continuous and discrete data using alternative models. This may be relatively easy for exponential models because, like power-law models, they possess a single parameter. Other alternative models such as log-normal models and exponentially modified power-law models may be computationally more difficult to apply because they possess two parameters. Furthermore, once two or three parameters are included in the model, the number of possible models increases quickly and the additional freedom supplied by additional parameters must be taken into consideration. Therefore, these logistical issues should be considered prior to performing comparisons against numerous alternative models.

In addition to various doubly truncated distributions, methods have been introduced to utilize mixtures of multiple types of distributions (so called “cocktail models”) to fit power-law and power-law-like data (Holden and Rajaraman, 2012; van Rooij et al., 2013; Ma et al., 2015). This type of mixture model could allow the user to fit a larger proportion of the data than would be the case by attempting to fit regions of the distribution that are purely power-law, as was done here. However, as discussed above, the extra freedom associated with additional free parameters must be accounted for when using these methods.

A potential criticism of the *p*-value method for detecting a power-laws that was utilized herein and elsewhere (Clauset et al., 2009; Deluca and Corral, 2013; Alstott et al., 2014) is the role played by the number of samples in the distribution. Simply put, increasing the number of samples makes it harder to accept data as being power-law (easier to reject the power-law hypothesis). As the number of samples increases, small fluctuations away from power law become larger in the KS-statistic value relative to noise. Therefore, this *p*-value method does not lend itself to testing fit ranges via bootstrap sub-sampling, for instance. One possible method to address this sample size effect is to develop a different method for comparing empirical distributions to the power-law fits produced by MLE. Perhaps something like a weighted *R*^2^-value could be developed. Then, *R*^2^-values for alternative or null models could be compared to the *R*^2^ for the real data. However, how to properly weight the data in the *R*^2^ calculation remains unclear. Still, we believe this is a promising approach and we hope to pursue it in the future.

### 3.4. Software

The NCC MATLAB toolbox (see Supplementary Material, Timme, 2016) contains numerous functions to perform power-law fitting analyses. The functions in the toolbox can be generally divided into three types: specialized functions, macros, and demonstration scripts. We will discuss the functions in the general order which the analysis is presented above with demonstration scripts discussed with their corresponding functions.

The doubly truncated MLE power-law fit of continuous (methodology introduced by Deluca and Corral, 2013) or discrete data with known minimum and maximum truncation points is performed with the function plmle:

**» x = gendata(10000, {’powerlaw’, 1.5}); %
      Generate random data
» tau = plmle(x); % Perform the fit assuming no
        truncation**

The function plmle allows for variable double truncation, variable search range for the exponent τ (including 0 < τ ≤ 1), and for variable precision in the exponent solution (thereby controlling the stop criterion for the lattice search). The variable precision controls the stop criterion for the lattice search algorithm discussed above.

In order to compare the functionality of existing tools and the NCC Toolbox, we tested the performance of the plmle function against the corresponding function (plfit) from Clauset et al. (2009) in MATLAB. We generated 10 sets of 10^4^ power-law distributed data points (τ = 2) using the randht function from Clauset et al. (2009) and discretized them by rounding. On average, plfit took 33.5 s to fit each untruncated data set, whereas plmle took 0.1 s. Both functions performed significantly better when continuous data was used. On average, plfit took 0.013 s to fit each untruncated data set, whereas plmle took 0.005 s. This test was performed on a Windows laptop (64-bit) with a standard quadcore processor. We believe this substantial difference in computation time is due to the use of the lattice search algorithm in plmle. We were unable to compare performance on doubly truncated power-law data because plfit is unable to fit doubly truncated power-law data.

To aid with visualization of data, the function plplottool can be used to calculate and plot histograms for continuous or discrete data on log-log axes. It can also be used to plot continuous or discrete doubly truncated power-law fits. It allows the user to select the size of logarithmic bins for continuous data or when binning discrete data. It also allows the user to control numerous aspects of the appearance of the plot and it outputs data in an easy format for plotting in other applications.

**» x = gendata(10000, {’powerlaw’, 1.5}); %
      Generate random data
» tau = plmle(x); % Perform the fit assuming no
        truncation
» % Set fit parameters
» fitParams = struct; fitParams.tau = tau;
» plotdata = plplottool(x,’fitParams’,fitParams);
             % Plot**

Many aspects of the functionality of plplottool are demonstrated in the script demoplotting:

**» demoplotting**

The *p*-value calculation used to determine if a segment of data is fit by a power-law is carried out using the function pvcalc:

**» x = gendata(10000, {’powerlaw’, 1.5}); %
      Generate random data
» tau = plmle(x, ’xmin’, 10, ’xmax’, 50); %
        Fit for one section of data
» p = pvcalc(x, tau, ’xmin’, 10, ’xmax’, 50); %
      Perform *p*-value calculation**

In pvcalc, the user can control the number of random data sets that are generated, a threshold for what *p*-value to consider acceptable, and a threshold to halt the computation should the likelihood of finding meeting the *p*-value threshold be too low. Furthermore, pvcalc can return the standard deviation of the exponents from the power-law model fits used to calculate the *p*-value. This standard deviation can be used as a measure of the error of the exponent of the original fit (Deluca and Corral, 2013). Several features of pvcalc and its overall performance are demonstrated in the script demotruncdist:

**» demotruncdist**

The function plparams carries out a search of the fit ranges to find the largest range that can be fit by a power-law:

**» % Generate random data with a power-law region
» x = gendata(100000, {’truncated_powerlaw’,
      [1.5, 0.125, 10, 75]});
» [tau, xmin, xmax] = plparams(x) % Find the
                      power-law region
» % Plot the results
» fitParams = struct;
» fitParams.tau = tau; fitParams.xmin = xmin;
  fitParams.xmax = xmax;
» plplottool(x,’fitParams’,fitParams)**

The plparams function allows the user to control the number of power-law models to generate in calculating the *p*-value, the *p*-value threshold for accepting a region as being fit by a power-law, and the likelihood threshold to cease *p*-value calculation early to save computation time.

The fits of the avalanche size given duration data is performed by the function sizegivdurwls:

**» load sample_data.mat % Load the data
» Av = avprops(asdf2); % Find the avalanches
» % Perform the size given duration fit and plot
  (see Figure 5)
» SNZ = sizegivdurwls(Av.size, Av.duration,
  ’durmin’, 4, ’durmax’, 22, ’plot’)**

Note that the fit regions (minimum and maximum duration) are set via minimum duration and minimum occurrence cuts that can be controlled by the user. The function sizegivdurwls is also capable of returning the error in the fit exponent via the use of the standard Matlab function lscov for the least squares fitting algorithm.

Any type of power-law analysis discussed above can be carried out using the avpropvals function:

**» load sample_data.mat % Load the data
» Av = avprops(asdf2); % Find the avalanches
» % Perform the avalanche size fit (see Figure 2
  without cuts)
» [tau, xmin, xmax] = avpropvals(Av.size, ’size’,
                      ’plot’);
» % Perform the average size given duration fit
» [SNZ, xmin, xmax] = avpropvals({Av.size,
                      Av.duration}, ’sizgivdur’);**

All of the power-law fitting functionality discussed above is demonstrated in the script demoempdata:

**» demoempdata**

Note that demoempdata also demonstrates shape collapse analyses discussed below.

Finally, we include the function randomizeasdf2 to produce randomized null model data sets for comparison to results from real data:

**» load sample_data.mat % Load the data
» % Jitter the spikes using a normal distribution
  with std = 20 ms
» randasdf2 = randomizeasdf2(asdf2,’jitter’,
  ’stdtime’,20);
» Av = avprops(asdf2); randAv =
       avprops(randasdf2); »
» plplottool({Av.size; randAv.size});**

The function randomizeasdf2 is capable of jittering, swapping, Poisson randomizing, and wrapping asdf2 format data. The user can control the standard deviation of the jittering distribution.

## 4. Shape collapse

### 4.1. Background

**Figure 6:**
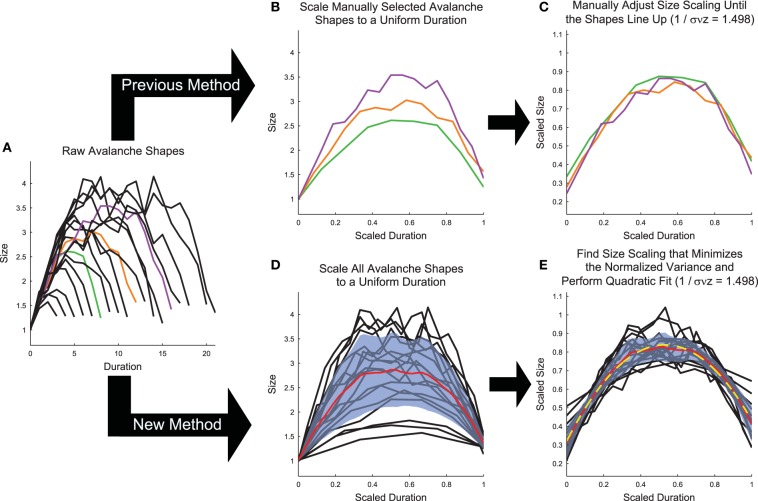
**Shape collapse calculation algorithm**. **(A)** Raw avalanche shapes are found by averaging the profile of all avalanches with a given duration. Only durations longer than three and with at least 20 examples were analyzed. In previous analyses (Friedman et al., 2012), **(B)** three avalanches are manually selected and scaled to uniform length (colors correspond to unscaled avalanches in **A**), then **(C)** the scaling parameter is manually adjusted until the shapes line up (Note, the same scaling is used in **C**,**E**). In the new method introduced herein, **(D)** all of the avalanches are scaled to a uniform length, each avalanche is linearly interpolated at 1000 points, and the variance is calculated (Standard deviation (blue fringe) shown instead of variance to make the error visible). **(E)** Then, the scaling parameter is found that minimizes the variance and the avalanches are fit (yellow dashed line) using a quadratic polynomial. Note, using the shape collapse analysis, we found 1∕σν*z* = 1.498. Using the average size given duration fit, we found 1∕σν*z* = 1.503 (Figure 5). This represents a difference of 0.3%. The data for this explanatory figure was taken from the cortical branching model.

If a neural system is in a critical state, in addition to exhibiting power-law size and duration distributions, the mean temporal profiles of avalanches should be identical across scales (Friedman et al., 2012). In other words, the profiles (Figures 1C, 6A) of long duration avalanches should have the same scaled mean shape as short avalanches. This phenomenon is also referred to as “shape collapse.” Specifically, the mean number of spiking neurons (*s*) at time *t* in an avalanche of duration *T* is related to the universal scaling function for the avalanche temporal profile *F* via Equation (13) (Friedman et al., 2012).

(13) $$\begin{array}{l} s\left(t,T\right)\propto T^{\gamma }F\left(t∕T\right) \end{array}$$

In Equation (13), γ is the scaling parameter that controls how much larger in size long duration avalanches are than short duration avalanches. Therefore, if the correct scaling parameter γ is chosen and if the system is close to criticality, when plotted on a scaled duration (i.e., *t*∕*T*) avalanches of all durations should produce the same mean profile when scaled via *s*(*t*, *T*)*T*^−γ^.

Using Equations (3, 13, and 14), it can be shown (Friedman et al., 2012) that 1∕σν*z* (see Equation 3) is related to the shape collapse scaling parameter γ via Equation (15).

(14) $$\begin{array}{lll} \langle S\rangle \left(T\right) & =\int_{0}^{T}s\left(t,T\right)dt\; & \; \end{array}$$

(15) $$\begin{array}{lll} \gamma & =\frac{1}{\sigma \nu z}-1\; & \; \end{array}$$

Therefore, it is possible to measure 1∕σν*z* using both the shape collapse and the average size given duration. The comparison between these values - which should be identical if the system is truly poised at a critical point - can be an important check of the criticality hypothesis. Note, for instance, in the sample model data set provided in the software toolbox, the average size given duration analysis produced 1∕σν*z* = 1.503 (Figure 5) and the shape collapse analysis produced 1∕σν*z* = 1.498 (see below, Figure 6E). This small difference of 0.3% is highly relevant for the criticality analysis in this model system.

While the shape collapse analysis has been applied previously to neural data (Friedman et al., 2012; Priesemann et al., 2013), we improved upon the methodology of previous analyses by automating the scaling parameter search process and by utilizing many more avalanche profiles. Previous shape collapses were performed manually and only included ~3 unique durations (Friedman et al., 2012) (Figure 6B). This was especially problematic given that the definition of the “good” shape collapse is very subjective and essentially relies on the researcher's opinion as to whether a few unique durations can be scaled to line up on top of each other (Figure 6C). Our shape collapse method quantified the quality of the shape collapse and automatically found the scaling parameter that produced the best possible collapse. Furthermore, it quantified the shape of the resulting collapse. Therefore, we did not assess if the data exhibited shape collapse, as has been done previously in a qualitative sense (Friedman et al., 2012), because developing a quantifiable method to determine if a particular data set exhibits shape collapse has proven difficult (though see Shaukat and Thivierge, 2016 for a recent attempt to do so). Rather, we feel that it is more appropriate to apply the shape collapse algorithm to the avalanches and interpret the resulting scaling parameter and shape parameters.

### 4.2. Improvements

Our shape collapse algorithm functioned as follows: We first removed avalanches with durations less than 4 and avalanches that occurred less than 20 times in a recording. We eliminated short avalanches because mean profiles for avalanches with durations less than 4 were only defined at 3 or fewer points, making the shape of the avalanche difficult to interpret. We eliminated avalanche durations with less than 20 occurrences to reduce the error of the mean size values for each point in the avalanche. Note that these were identical to the cuts applied to the avalanches during the power-law fitting analysis (see Section 3).

After applying cuts to the data, we calculated the average avalanche profile for each unique duration (Figure 6A). We then scaled all durations to length 1 (i.e., *t*∕*T*; Figure 6D). Next, we linearly interpolated each avalanche at 1000 points along the scaled duration and we calculated the variance across the avalanche profiles at the interpolated points. We then calculated the shape collapse error as the mean variance divided by the square span of the avalanche shapes, where the span equaled the maximum profile value minus the minimum profile value. We then performed an automated lattice search of scaling parameter values to find the scaling parameter that minimized the shape collapse error (Figure 6E). The lattice search began by searching the interval 1 ≤ γ ≤ 5 at increments of 0.1. The lattice search proceeded to a precision of 10^−3^ via three steps (identical methodology to the power-law MLE exponent lattice search; see Section 3.2).

Following the automated shape collapse, we fit the scaled avalanches (using the 1000 linearly interpolated points) with a quadratic polynomial using least squares fitting via the standard Matlab function polyfit (*f*_*fit*_(*t*∕*T*)) (Figure 6E). We then calculated the absolute curvature of the fit (Equation 16) at each interpolated point.

(16) $$\begin{array}{l} c\left(t∕T\right)=\frac{\left|f_{fit}^{\prime \prime }\left(t∕T\right)\right|}{{\left(1+f_{fit}^{\prime }{\left(t∕T\right)}^{2}\right)}^{3∕2}} \end{array}$$

We found it useful to quote the average absolute curvature to quantify the shape of the collapse. It is possible for avalanches to be flat and still produce data collapses with meaningful scaling parameters, but we wished to be able to quantify the flatness of the shape collapses. Note that avalanches could produce non-symmetric shapes or shapes better fit by a different function. Though, in our experience with neural avalanches, quadratic fits generally function well and lend themselves easily to a calculation of the curvature. We suggest that other researchers consider whether another function would be a more appropriate fit for their system.

### 4.3. Discussion

Our shape collapse method represents a significant improvement over existing shape collapse analysis methods because it is automated, it takes many avalanches into account, and it is quantitative. Still, we were unable to develop a method that could determine when a given data set exhibited shape collapse or to produce a quantifiable metric that could be used to judge the quality of the fit. We attempted to quantify the quality of the shape collapse itself by examining the shape collapse error values. However, we found that the shape collapse error value itself was not easy to compare between data sets, primarily due to the normalization of the error by the span of the avalanches. The span was a useful normalization factor within a data set because it set a scale for the avalanches that was unbiased by the scaling parameter. Other normalization metrics, such as the mean value of the avalanches, were found to produce unsatisfactory shape collapses due to biases with the scaling parameter. However, the span value was extremely noisy across data sets because it depended primarily on the highest and lowest sampled avalanche duration that survived the occurrence cuts.

Recently, a new method was introduced to quantify the quality of a shape collapse (Shaukat and Thivierge, 2016). This method used filtering operations to smooth shapes, a time rescaling, and an *F*-test to determine if the shapes reliably overlapped under permutations. However, to the best of our understanding, that method is unable to automatically perform the shape collapse or measure the scaling parameter, which are essential components of the shape collapse analysis. Furthermore, the methods introduced in Shaukat and Thivierge (2016) are significantly more complicated than the method discussed in this paper. In the future, we hope to develop better methods to quantify the quality of the shape collapse.

### 4.4. Software

The NCC MATLAB toolbox (see Supplementary Material, Timme, 2016) contains functions to perform the shape collapse analyses. First, avalanche profiles are found by averaging the shapes of all avalanches with the same durations via the function avgshapes:

**» load sample_data.mat % Load the data
» Av = avprops(asdf2); % Find the avalanches
» avgProfiles = avgshapes(Av.shape, Av.duration,
                ’cutoffs’, 4, 20);**

The avgshapes function allows the user to select from numerous methods for limiting which duration avalanches are considered, include a minimum duration cut (e.g., 4) and a minimum occurrence cut (e.g., 20), as shown above.

Next, the shape collapse is performed by the function avshapecollapse to yield a value for 1∕σν*z* (see Figure 6E):

**» [SNZsc, secondDrv, range, errors] =
  avshapecollapse(avgProfiles, ’plot’);**

When performing the shape collapse with avshapecollapse, the user can vary the precision of the lattice search goal (i.e., the stop criterion), the bounds for the exponent search, and the number of interpolation points along the average avalanche profiles.

The error in the shape collapse exponent can be estimated with the function avshapecollapsestd:

**» sigmaSNZsc = avshapecollapsestd(avgProfiles);**

The error estimate for the shape collapse exponent is calculated by a bootstrap routine of sampling the avalanches, performing the collapse, and calculating the standard deviation of the resulting shape collapse exponents. The function avshapecollapsestd allows for the same user controlled parameters as avshapecollapse, as well as user control over the number of samples of the average avalanche profiles and the number of sampling trials to perform.

Finally, along with the power-law fitting routines discussed above, the shape collapse routine is demonstrated in the script demoempdata:

**» demoempdata**

## 5. Complexity

### 5.1. Background

Our calculation of complexity closely followed the original description in Tononi et al. (1994), with a few slight alterations. Our primary contribution to the complexity calculation was a system state sub-sampling bias correction algorithm not previously described in the literature, to the best of our knowledge. Before discussing this improvement, we will first discuss the basic complexity calculation itself. We calculated the complexity in a system of *N* spiking neurons (call this system *X*). We were able to exploit the discrete nature of the neural variables because we focused on the complexity of a system of neurons, though a system of electrodes recording LFP times could also be analyzed using these methods. Other studies have measured neural complexity in systems with continuous variables and require additional attention (Tononi et al., 1994; van Putten and Stam, 2001; Burgess et al., 2003; van Cappellen van Walsum et al., 2003). The entropy of a system of *N* spiking neurons is given by Equation (17) (Cover and Thomas, 2006).

(17) $$\begin{array}{l} H\left(X\right)=-\underset{i}{\sum }p\left(x_{i}\right)\log\left(p\left(x_{i}\right)\right) \end{array}$$

In Equation (17), *x*_*i*_ is a joint state of all *N* neurons at a given time bin and the base of the logarithm is 2 to yield information results in units of bits. In our analysis, the probability of a given joint state of neurons *p*(*x*_*i*_) was found by counting the number of occurrences of a given state throughout a recording and dividing by the total number of states. We assumed the probability distributions *p*(*x*_*i*_) were stationary throughout the recording.

By comparing the joint entropy of a group of neurons to the sum of their individual entropies, it is possible to measure the degree to which the activities of the neurons are coordinated. This measure is referred to as the integration (the integration has previously been referred to as the total correlation Watanabe, 1960; Tononi et al., 1994; Timme et al., 2014). When considering a subset of neurons, we note the *j*^*th*^ unique set of *k* neurons as $X_{j}^{k}$. So, $X_{j}^{1}$ would refer to the *j*^*th*^ neuron alone, but $X_{j}^{3}$ would refer to the *j*^*th*^ unique set of three neurons. Using this notation, the integration of the *j*^*th*^ set of *k* neurons is given by Equation (18).

(18) $$\begin{array}{l} I\left(X_{j}^{k}\right)=\left(\underset{j^{\prime }\in k}{\sum }H\left(X_{j^{\prime }}^{1}\right)\right)-H\left(X_{j}^{k}\right) \end{array}$$

Using the integration, the complexity is given by Equation (19) (Tononi et al., 1994; van Putten and Stam, 2001).

(19) $$\begin{array}{l} C_{N}\left(X\right)=\frac{1}{N}\overset{N}{\underset{k=2}{\sum }}\left[\left(\frac{k-1}{N-1}\right)I\left(X\right)-{\langle I\left(X_{j}^{k}\right)\rangle }_{j}\right] \end{array}$$

In the data analysis, the average subset integration (${\langle I\left(X_{j}^{k}\right)\rangle }_{j}$) was calculated at each possible value of *k* from 2 to *N*. Given the large number of permutations for most values of *k* for large numbers of *N*, it was often not possible to exhaustively sample all permutations (i.e., all unique sets of *k* variables). If the number of possible permutations was less than or equal to 100, all possible permutations were exhaustively calculated and averaged. In all other cases, 100 permutations were randomly chosen and the integration for these subsets were averaged. These calculations yielded subset average integration curves for all values of *k*. The complexity is then the difference between the integration curves across all subset sizes and the linear approximation for the integration curve based on the integration for the whole system, normalized by the total number of neurons (Equation 19).

**Figure 7:**
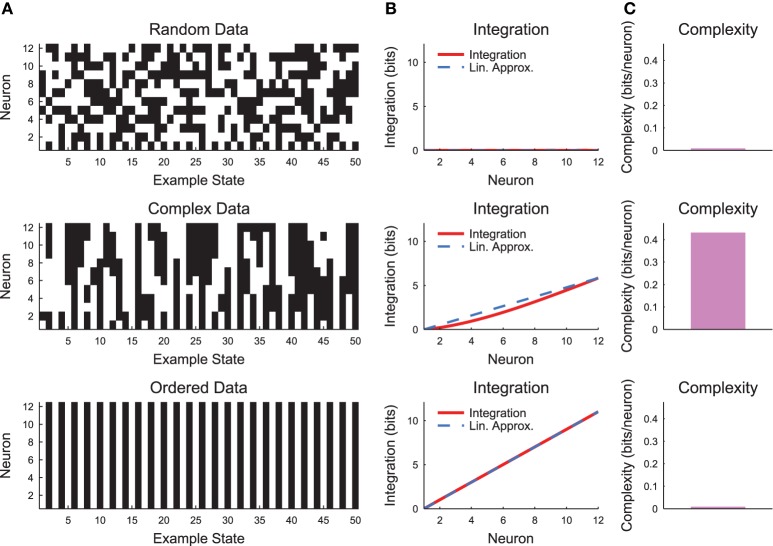
**Neural complexity**. **(A)** Short segments of example spike rasters for three types of chain model data [see Section 2.3, *c* = 0 (random), *c* = 0.8 (complex), *c* = 1 (ordered)]. **(B)** Integration curves with linear approximations for different subset sizes. Note that random data shows no integration, while ordered data shows high integration. Complex data shows high integration that varies non-linearly with subset size. **(C)** Complexity values. Only the complex data shows non-zero complexity.

The complexity as expressed in Equation (19) can be difficult to interpret. Therefore, it is helpful to evaluate the complexity in a simple system such as a small chain model (see Section 2.3, Figure 7). Complexity requires some degree of coordinated variability across many scales in the system. In Figure 7, we show three types of models: a random model, a complex model, and an ordered model. The behaviors of the models are apparent from a brief segment of representative spike rasters (Figure 7A). The random data contain no interactions, while the ordered data contain no variability. The complex data show some balance between these states. When the integration curves are plotted (Figure 7B), the random data produce zero integration, while the ordered data produce high integration. However, the complex data produce a non-linear integration curve, suggesting varying interactions across scales and non-zero complexity.

The model used to generate example results for Figure 7 was small and well defined (i.e., the precise joint probability distributions were defined). Conversely, neural data typically include many more variables and the joint probability distribution must be estimated from available observations. Unfortunately, these two realities of neural data (many variables and limited observations) could produce sub-sampling bias in the following sense. The integration calculation (Equation 18) requires comparisons between entropies of individual variables and joint entropies of large sets of variables. These two calculations experience very different levels of state sub-sampling bias, thus making state sub-sampling bias in the integration calculation likely for data sets with many variables (see below for a demonstration of this effect). Previous analyses have dealt with this issue by making assumptions about the underlying structure of the data (e.g., converting neural signals to Gaussian distributions Tononi et al., 1994). We sought a different approach to address possible state sub-sampling bias.

### 5.2. Improvements

**Figure 8:**
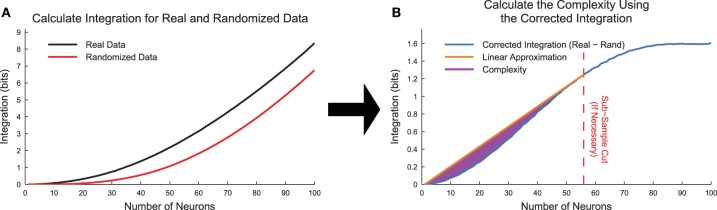
**Complexity calculation algorithm**. **(A)** Integration curves were calculated for each subset size for both real and randomized data. Frequently, the randomized data produced non-zero integration curves, indicating the presence of state sub-sampling bias. **(B)** To account for the sub-sampling bias, we calculated the corrected integration by subtracting the randomized integration curve from the real integration curve. In cases where the corrected integration curve was not uniformally concave up due to sub-sampling, we utilized an automated method to apply a cut and thereby limit the system size based on the available data. The complexity (Equation 21) is represented by the area between the linear approximation and the corrected integration curve. The data for this explanatory figure was taken from the cortical branching model.

To test for the effects of state sub-sampling, we also calculated the integration curves for randomized data. These randomized data were created by randomly placing a neuron's spikes with equal likelihood at all time points. This process essentially converted each neuron into a random Poisson process with firing rate matching the original data. Using data from the cortical branching model, we found that the integration was non-zero for large subset sizes (Figure 8A). This contradicted expectations because in a totally random system, the integration should be zero. This effect was caused by sub-sampling of the joint distribution *p*(*x*_*i*_). We can better understand this effect by examining the integration. In Equation (18), the individual entropies $H\left(X_{j^{\prime }}^{1}\right)$ are relatively well sampled because they depend on only individual binary neurons. However, the joint entropy $H\left(X_{j}^{k}\right)$ is poorly sampled for large *k*. For instance, for a subset of *k* = 50 neurons, the system can occupy 2^50^ ≈ 10^15^ possible states, which cannot be experimentally sampled from a biological system. This poor sampling leads to an underestimation of the number of states available to the system, and therefore an underestimation of the joint entropy of the system ($H\left(X_{j}^{k}\right)$). In an attempt to correct for this sub-sampling bias, we calculated a corrected integration by subtracting the randomized integration from the original integration (Figure 8B, Equation 20).

(20) $$\begin{array}{l} {\langle I\left(X_{j}^{k}\right)\rangle }_{j,Cor}={\langle I\left(X_{j}^{k}\right)\rangle }_{j,Real}-{\langle I\left(X_{j}^{k}\right)\rangle }_{j,Rand} \end{array}$$

When we attempted to calculate the complexity using the corrected integration (note that $I\left(X\right)=I\left(X_{1}^{N}\right)$), we found that some data sets produced corrected integration curves that were concave down (i.e., negative second derivative in *k*) for high *k* values. In a fully sampled system, the integration curves should be uniformly concave up, so this behavior was an indication of sub-sampling bias even when using the corrected integration.

To correct for sub-sampling bias in the corrected integration, we used an automated method to find the first point (call it *k*_*max*_) on the corrected integration curve that produced the largest slope line when connected to the point (*k* = 1, *I* = 0). To perform this search, we utilized the standard Matlab function findpeaks. In some cases, *k*_*max*_ was found to equal *N*. In other cases all values with *k* > *k*_*max*_ were removed from the integration curve. It was assumed that the complexity calculation could only be accurately sampled up to size *k* = *k*_*max*_. Furthermore, we normalized the complexity by dividing by *k*_*max*_. This method resulted in a new corrected version of the complexity (Figure 8, Equation 21).

(21) $$\begin{array}{r} C_{N,Cor}\left(X\right)=\\ \frac{1}{k_{max}}\overset{k_{max}}{\underset{k=1}{\sum }}\left[\left(\frac{k-1}{k_{max}-1}\right){\langle I\left(X_{j}^{k_{max}}\right)\rangle }_{j,Cor}-{\langle I\left(X_{j}^{k}\right)\rangle }_{j,Cor}\right] \end{array}$$

In addition to utilizing the corrected complexity in Equation (21), we also found it useful to only included time bins where at least one neuron fired in the calculations. In other words, we found it useful to calculate the complexity of the avalanches. We found that this controlled for biases associated with different numbers of avalanches in a data set.

### 5.3. Discussion

We would like to note that because neural avalanches incorporate information about interactions through time, while the complexity calculation does not, they are not trivially related. For instance, a neural avalanche is defined in part by the number of consecutive time bins during which activity was present in the network. Conversely, the complexity calculation only incorporates the total number of times a given state occurs in a time series, not the order or temporal relationships between the states.

While we feel our state sub-sampling correction algorithm is a significant improvement over previous methods that require assumptions about the underlying data (Tononi et al., 1994) or simply ignoring state sub-sampling, we recognize that this method could still suffer from sub-sampling bias. The subject of sub-sampling in entropy calculations has been addressed previously (e.g., Strong et al., 1998; Nemenman et al., 2004), but, to the best of our knowledge, no universal solution exists to the sub-sampling problem in entropy calculations. A possible alternative method would be to only consider subsets of size *k* such that the number of possible states that the subset could occupy would be much less than the total number of observations. In other words, set *k*_*max*_ such that $2^{k_{max}}\ll N_{time}$. This method would have the effect of restricting the analysis to the left portion of the integration curve prior to where the randomized integration curve deviates substantially from zero (see Figure 8). We feel this would be a useful method, but we note that it would require the choice of a parameter to set the ratio of possible system states to the number of observed states. Furthermore, the sub-sampling bias will probably be dependent upon which neurons are selected for the subset, with some low entropy sets not exhibiting sub-sampling bias, while other high entropy sets exhibiting sub-sampling bias at smaller subset sizes. The method we introduced automatically detects the point where sub-sampling bias significantly impacts the integration calculation in a parameter free fashion. In the future, we look forward to developing improved methods for addressing state sub-sampling in the complexity calculation.

### 5.4. Software

The NCC MATLAB toolbox (see Supplementary Material, Timme, 2016) contains functions to perform the complexity analyses. In general, the complexity analyses utilize raster formatted data (see Section 2.4). Once the data is represented as a raster, the complexity can be calculated with the function complexity:

**» load sample_data.mat; raster =
  asdf2toraster(asdf2);
» % Perform the complexity analysis without the
sub-sampling correction
» Cn = complexity(raster, 100); % Use 100 samples
       of *j* for each size *k*
» % Perform the complexity analysis with the
  sub-sampling correction
» Cn = complexity(raster, 100, ’subsampcorrect’);**

The full functionality of the complexity software is demonstrated in the script democomplexity (see Figure 8):

**» democomplexity**

The simple chain model and the related complexity calculation example shown in Figure 7 can be generated using the script complexityexamples:

**» complexityexamples**

## 6. Main findings

In this work, we have described several advances in analyses of power laws, neural criticality, and neural complexity. First, we improved upon previous power-law fitting methods by developing an automated method to find power-law regions in probability distributions. This is especially relevant given the possibility for biases on either end of a purported power-law distribution. Second, we developed an automated method for performing avalanche shape collapse. This is an important contribution because it increases repeatability in shape collapse analyses. Third, we developed new tools for measuring neural complexity that account for state sub-sampling (i.e., short recording lengths in comparison to the possible number of states the system could occupy). Given the importance of quantifying complex behavior and the reality of data gathering limitations, these advances are noteworthy. Finally, all of the analyses described in this article can be carried out using the freely available NCC MATLAB software toolbox.

## Author contributions

NM and NT developed the new analysis methods, wrote the software, and wrote the manuscript. NB, MR, and EL contributed to the software and the manuscript. JB oversaw the project and contributed to the manuscript.

## Funding

This research was supported by National Science Foundation (http://www.nsf.gov) grants 090813 (JB), 1058291 (JB), CNS-0521433 (Indiana University computing resources), and CNS-0723054 (Indiana University computing resources). This research was also supported by the Mind Science Foundation. Also, via the use of computing resources at Indiana University, this research was supported in part by Lilly Endowment, Inc., through its support for the Indiana University Pervasive Technology Institute, and in part by the Indiana METACyt Initiative. The funders had no role in study design, data collection and analysis, decision to publish, or preparation of the manuscript.

### Conflict of interest statement

The authors declare that the research was conducted in the absence of any commercial or financial relationships that could be construed as a potential conflict of interest.
